# Betel Nut Chewing Increases the Risk of Metabolic Syndrome and Its Components in a Large Taiwanese Population Follow-Up Study Category: Original Investigation

**DOI:** 10.3390/nu14051018

**Published:** 2022-02-28

**Authors:** Ya-Chin Huang, Jiun-Hung Geng, Pei-Yu Wu, Jiun-Chi Huang, Szu-Chia Chen, Jer-Ming Chang, Hung-Chun Chen

**Affiliations:** 1 Department of Preventive Medicine, Kaohsiung Municipal Ta-Tung Hospital, Kaohsiung Medical University, Kaohsiung 801, Taiwan; jasimine0603@gmail.com; 2 Department of Occupational & Environmental Medicine, Kaohsiung Medical University Hospital, Kaohsiung Medical University, Kaohsiung 807, Taiwan; 3 Department of Urology, Kaohsiung Municipal Siaogang Hospital, Kaohsiung Medical University, Kaohsiung 812, Taiwan; u9001090@gmail.com; 4 Department of Urology, Kaohsiung Medical University Hospital, Kaohsiung Medical University, Kaohsiung 807, Taiwan; 5 Department of Internal Medicine, Kaohsiung Municipal Siaogang Hospital, Kaohsiung Medical University, Kaohsiung 812, Taiwan; wpuw17@gmail.com (P.-Y.W.); karajan77@gmail.com (J.-C.H.); 6 Department of Internal Medicine, Division of Nephrology, Kaohsiung Medical University Hospital, Kaohsiung Medical University, Kaohsiung 807, Taiwan; 7 Faculty of Medicine, College of Medicine, Kaohsiung Medical University, Kaohsiung 807, Taiwan; 8 Research Center for Environmental Medicine, Kaohsiung Medical University, Kaohsiung 807, Taiwan

**Keywords:** betel nut chewing, metabolic syndrome, metabolic syndrome components, Taiwan Biobank

## Abstract

Betel nut chewing is a popular habit in Taiwan, and it is associated with adverse metabolic effects. The aim of this study was to investigate correlations between betel nut chewing with metabolic syndrome (MetS) and its components in a longitudinal study using data from the Taiwan Biobank. A total of 121,423 participants were included in the baseline study, and 27,002 received follow-up examinations after a median of 4 years. The association between betel nut chewing and MetS was analyzed using multiple logistic regression after controlling for confounders. The baseline prevalence of MetS was 22.5%. Multivariable analysis showed that a history of chewing betel nut was significantly associated with baseline MetS (odds ratio (OR) = 1.629; 95% confidence interval (CI) = 1.535 to 1.730, *p* < 0.001) and five components of MetS in all participants. A long history of chewing betel nut (per 1 year; OR = 1.008; 95% CI = 1.004 to 1.013, *p* < 0.001) was associated with baseline MetS, abdominal obesity, hypertriglyceridemia and low high-density lipoprotein (HDL) cholesterol. In addition, high cumulative dose (per 1 year × frequency × daily score; OR = 1.001; 95% CI = 1.001–1.002; *p* < 0.001) was significantly associated with baseline MetS. At the end of the follow-up, a history of chewing betel nut (OR = 1.352; 95% CI = 1.134 to 1.612, *p* = 0.001) was significantly associated with MetS and its components including abdominal obesity, hypertriglyceridemia and low HDL-cholesterol in the participants without baseline MetS. In addition, a longer history of betel nut chewing was associated with MetS (per 1 year; OR = 1.021; 95% CI = 1.008 to 1.035, *p* = 0.002), abdominal obesity and hypertriglyceridemia at follow-up. However, cumulative dose (*p* = 0.882) was not significantly associated with follow-up MetS. Chewing betel nut and a long history of betel nut chewing were associated with baseline MetS and its components. In the participants without MetS at baseline, chewing betel nut and a long history of chewing betel nut were associated with the development of MetS after 4 years of follow-up. However, a cumulative dose of betel nut chewing was not associated with follow-up MetS. Betel nut chewing cessation programs are important to reduce the incidence of MetS in Taiwan.

## 1. Introduction

Metabolic syndrome (MetS) is an expanded definition of insulin resistance and also known as syndrome X which was first described by Gerald M. Raevan in 1988 [1]. It encompasses a cluster of cardiovascular risk factors including diabetes mellitus (DM), prediabetes, high cholesterol level, high blood pressure (BP) and abdominal obesity [2]. The Asia-Pacific region is facing an epidemic of MetS, where nearly one in five adults are affected [3]. MetS is associated with higher risks of atherosclerotic cardiovascular disease [4,5], type 2 DM [6,7], ischemic stroke [8,9], sleep apnea [10], polycystic ovary disease [11], non-alcoholic fatty liver disease [12] and cancer [13]. Therefore, elucidating the factors associated with an increased risk of developing MetS is an important issue.

Betel nut chewing is the fourth most abused substance globally [14], with reported lifetime chewing rates of 15.6% in Taiwan and 43.6% in Nepal [15]. The International Agency for Research on Cancer has shown that betel nut chewing is associated with cancers of the oral cavity, pharynx and esophagus [16]. In addition, epidemiological studies have reported that betel nut chewing is associated with general and central obesity [17], hypertension [18], chronic kidney disease [19], cardiovascular disease [20,21,22], all-cause mortality [20,22] and liver cirrhosis [23]. Moreover, betel nut chewing has been associated with glucose intolerance and type 2 DM in animal and human studies [24,25], and other studies have demonstrated a relationship between betel nut chewing and MetS [22,26,27,28]. Evidence indicates that certain components of alkaloids in areca nut and areca-nut-derived nitrosamines could increase the appetite and block insulin signaling in adipocytes and may have important roles in the association with MetS [28,29]. However, few studies have investigated the association between the cumulative effect of betel nut chewing and the components of MetS in a large-scale follow-up study. Therefore, the aim of this study was to investigate correlations between betel nut chewing with MetS and its components in a longitudinal study with a large cohort of participants enrolled in the Taiwan Biobank (TWB).

## 2. Materials and Methods

### 2.1. Ethics Statement

This study was conducted following the tenets of the Declaration of Helsinki, and approval was granted by the Institutional Review Board of Kaohsiung Medical University Hospital (KMUHIRB-E(I)-20210058). Ethical approval for the TWB was granted by the Institutional Review Board on Biomedical Science Research, Academia Sinica, Taiwan and the Ethics and Governance Council of the TWB. Written informed consent was obtained from all of the participants.

### 2.2. Taiwan Biobank

**Figure 1:**
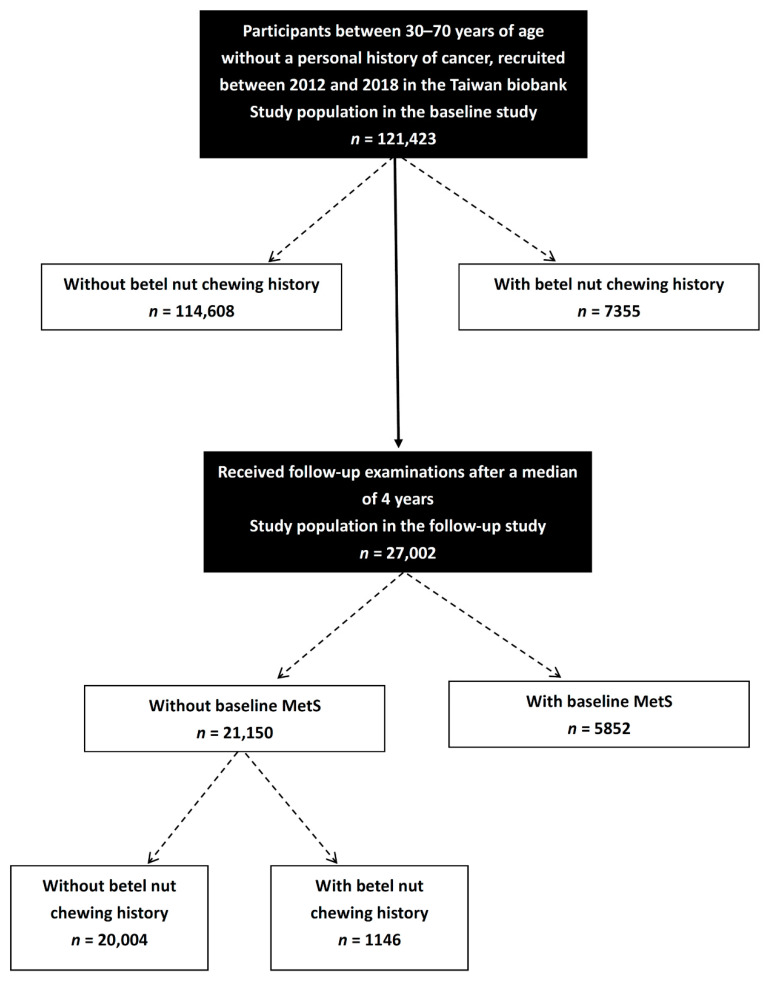
Flowchart of study population.

Due to the aging population and extended average life span in Taiwan, the Ministry of Health and Welfare established the TWB with the aim of promoting health care and preventing chronic diseases. The TWB contains lifestyle, genetic and medical data of community-based volunteers aged 30 to 70 years with no history of cancer [30,31]. On enrollment, the volunteers receive an in-person interview and physical examination and blood samples are taken. During the physical examination, data on waist circumference (WC), body height, body weight and body mass index (kg/m^2^) are recorded. During the in-person interview, personal information including age, sex, personal and family medical histories (such as DM and hypertension), lifestyle factors (such as histories of smoking and alcohol consumption) and diet are recorded through a questionnaire. A total of 121,423 participants were included in the baseline study. Of these participants, 27,002 received follow-up examinations after a median of 4 years (Figure 1).

### 2.3. Collection of Study Variables

In addition to the data detailed above, we also collected data on exercise. For the purposes of this study, regular exercise was defined as performing 30 min of physical activity three times a week, including but not limited to computer-based exercise activities, cycling, hiking, yoga, aerobics, running, playing a sport, swimming and cycling. However, occupational-related activities were not included as exercise.

The following laboratory data were also recorded: fasting glucose, triglycerides, total cholesterol, high-density lipoprotein (HDL)-cholesterol, low-density lipoprotein (LDL)-cholesterol, estimated glomerular filtration rate (eGFR) and uric acid. The eGFR was calculated using the four-variable Modification of Diet in Renal Disease study equation [32].

### 2.4. Definition of MetS

In this study, MetS was defined according to the NCEP-ATP III guidelines [33] and modified criteria for Asians [34] as meeting ≥ 3 of the following criteria: (1) abdominal obesity (WC ≥ 80 cm for women and ≥90 cm for men); (2) triglyceride concentration ≥ 150 mg/dL; (3) HDL-cholesterol concentration < 50 mg/dL for women and <40 mg/dL for men; (4) systolic BP ≥ 130 mmHg, diastolic BP ≥ 85 mmHg, diagnosed hypertension, or receiving hypertension treatment; (5) hyperglycemia, defined a fasting whole-blood glucose concentration ≥ 100 mg/dL or diagnosed DM.

### 2.5. Assessment of Betel Nut Chewing

The participants were asked ‘Have you ever chewed betel nut?’ If they answered ‘Yes’, then they were asked to answer the following questions:

1. ‘How many years have you chewed betel nut?’
2. ‘How often do you chew betel nut?’ (frequency)
  1–3 days/month (score = 1);
  1, 2 days/week (score = 2);
  3–5 days/week (score = 3);
  Every day (score = 4).
3. ‘How many quid a day?’ (daily amount)
  <10 quids (score = 1);
  10–20 quids (score = 2);
  21–30 quids (score = 3);
  ≥31 quids (score = 4).
4. Cumulative dose = years of chewing betel nut × frequency score × daily score.

### 2.6. Statistical Analysis

Data are presented as the mean ± standard deviation or *n* (%). Differences between categorical variables were examined using the chi-square test, and differences between continuous variables were examined using the independent *t*-test. Associations between chewing betel nut with MetS and its components were examined using multivariable logistic regression analysis. One-way analysis of variance was used for multiple comparisons among the study groups according to the number of MetS components. A *p*-value of less than 0.05 was considered to indicate a statistically significant difference. All statistical analyses were conducted using SPSS version 19.0 for Windows (SPSS Inc., Chicago, IL, USA).

## 3. Results

The mean age of the 121,423 enrolled participants was 49.9 ± 11.0 years old and they included 43,636 males and 77,787 females. The participants were stratified into two groups according to baseline MetS (−) (*n* = 94,043; 77.5%) or MetS (+) (*n* = 27,380; 22.5%).

### 3.1. Comparison of Clinical Characteristics among the Participants according to Baseline MetS

A comparison of the clinical characteristics between the participants without or with baseline MetS is shown in Table 1. Compared to the participants without MetS, those with MetS were older, predominantly male, had higher prevalence rates of DM and hypertension, higher prevalence rates of betel nut chewing, smoking and alcohol drinking histories, higher BMI, higher WC, higher fasting glucose, higher triglycerides, higher total cholesterol, lower HDL-cholesterol, higher LDL-cholesterol, lower eGFR and high uric acid.

### 3.2. Association between Betel Nut Chewing History with Baseline MetS and Its Components in All Participants

Table 2 shows the association of betel nut chewing history with baseline MetS and its components in all study participants (*n* = 121,423) using multivariable logistic regression analysis. In the multivariable analysis, adjusted for betel nut chewing history, age, gender, smoking history, alcohol history, regular exercise habit, total cholesterol, LDL-cholesterol, eGFR and uric acid), betel nut chewing history (odds ratio (OR) = 1.629; 95% confidence interval (CI) = 1.535 to 1.730; *p* < 0.001), old age (*p* < 0.001), female sex (*p* < 0.001), smoking history (*p* < 0.001), no regular exercise habits (*p* < 0.001), high total cholesterol (*p* < 0.001), low LDL-cholesterol (*p* < 0.001), high eGFR (*p* < 0.001) and high uric acid (*p* < 0.001) were significantly associated with baseline MetS.

Further, regarding MetS components, betel nut chewing history was significantly associated with baseline abdominal obesity (OR = 1.550; 95% CI = 1.465 to 1.640; *p* < 0.001), baseline hypertriglyceridemia (OR = 1.488; 95% CI = 1.399 to 1.582; *p* < 0.001), baseline low HDL-cholesterol (OR = 1.529; 95% CI = 1.435 to 1.630; *p* < 0.001), baseline hyperglycemia (OR = 1.222; 95% CI = 1.151 to 1.297; *p* < 0.001) and baseline high BP (OR = 1.139; 95% CI = 1.075 to 1.206; *p* < 0.001) in all study participants.

After exclusion of participants with smoking and/or alcohol drinking history (*n* = 85,705), we still found that betel nut chewing history (OR = 1.903; 95% CI = 1.472 to 2.459; *p* < 0.001) was significantly associated with baseline MetS.

### 3.3. Association between Duration of Chewing Betel Nut with Baseline MetS and Its Components in Study Participants with Betel Nut Chewing History

Table 3 shows the association of betel nut chewing years with baseline MetS and its components in study participants with betel nut chewing history (*n* = 7355) using multivariable logistic regression analysis. In the multivariable analysis, adjusted for duration of betel nut chewing, age, gender, smoking history, alcohol history, regular exercise habit, total cholesterol, LDL-cholesterol, eGFR and uric acid), a long duration of chewing betel nut (per 1 year; OR = 1.008; 95% CI = 1.004 to 1.013; *p* < 0.001), old age (*p* < 0.001), alcohol drinking history (*p* = 0.031), no regular exercise habits (*p* < 0.001), high total cholesterol (*p* < 0.001), low LDL-cholesterol (*p* < 0.001) and high uric acid (*p* < 0.001) were significantly associated with a low baseline MetS in the participants with a history of chewing betel nut. In addition, high cumulative dose (per 1 year × frequency × daily score; OR = 1.001; 95% CI = 1.001–1.002; *p* < 0.001) was significantly associated with baseline MetS.

Further, regarding MetS components, a long duration of chewing betel nut was significantly associated with baseline abdominal obesity (per 1 year; OR = 1.006; 95% CI = 1.001 to 1.010; *p* = 0.009), baseline hypertriglyceridemia (per 1 year; OR = 1.011; 95% CI = 1.006 to 1.016; *p* < 0.001) and baseline low HDL-cholesterol (per 1 year; OR = 1.012; 95% CI = 1.007 to 1.017; *p* < 0.001) in the participants with a history of chewing betel nut but not with baseline hyperglycemia (*p* = 0.283) and baseline high BP (*p* = 0.999).

We further performed comparisons of baseline MetS, five components of MetS and the numbers of MetS according to quartile of cumulative dose of betel nut chewing in study participants with betel nut chewing history (Table S1), and found that there were significant trends for stepwise increases in the prevalence of baseline MetS (*p* < 0.001), baseline abdominal obesity (*p* < 0.001), baseline hypertriglyceridemia (*p* = 0.001), baseline low HDL-cholesterol (*p* < 0.001), baseline hyperglycemia (*p* = 0.001), baseline high blood pressure (*p* = 0.019) and the numbers of baseline MetS (*p* < 0.001) corresponding to quartile of cumulative dose of betel nut chewing.

We further evaluated the association between personal habits of alcohol drinking, betel nut chewing and smoking history with baseline MetS, and found there was no significant difference among personal habits with baseline MetS.

### 3.4. Association between Betel Nut Chewing History with Follow-Up MetS and its Components in Follow-Up Participants without Baseline MetS

Table 4 shows the association of betel nut chewing history with follow-up MetS and its components in follow-up participants without baseline MetS (*n* = 21,150) using multivariable logistic regression analysis. In the multivariable analysis, adjusted for betel nut chewing history, age, gender, smoking history, alcohol history, regular exercise habit, total cholesterol, LDL-cholesterol, eGFR and uric acid), betel nut chewing history (OR = 1.352; 95% CI = 1.134 to 1.612; *p* = 0.001), old age (*p* < 0.001), female sex (*p* < 0.001), smoking history (*p* = 0.025), no regular exercise habits (*p* < 0.001), low total cholesterol (*p* < 0.001), high LDL-cholesterol (*p* < 0.001), high eGFR (*p* < 0.001) and high uric acid (*p* < 0.001) were significantly associated with follow-up MetS.

Further, regarding MetS components, betel nut chewing history was significantly associated with follow-up abdominal obesity (OR = 1.197; 95% CI = 1.035 to 1.384; *p* = 0.015), follow-up hypertriglyceridemia (OR = 1.326; 95% CI = 1.130 to 1.556; *p* = 0.001) and follow-up low HDL-cholesterol (OR = 1.345; 95% CI = 1.113 to 1.626; *p* = 0.002) in follow-up participants without baseline MetS but not with follow-up hyperglycemia (*p* = 0.083) and follow-up high BP (*p* = 0.980).

After exclusion of participants with smoking and/or alcohol drinking history (*n* = 15,478), betel nut chewing history (*p* = 0.164) was not significantly associated with follow-up MetS.

### 3.5. Association between Duration of Chewing Betel Nut with Follow-Up MetS and Its Components in Follow-Up Participants without Baseline MetS with a Betel Nut Chewing History

Table 5 shows the association of betel nut chewing years with follow-up MetS and its components in follow-up participants without baseline MetS with betel nut chewing history (*n* = 1146) using multivariable logistic regression analysis. In the multivariable analysis, adjusted for duration of betel nut chewing, age, gender, smoking history, alcohol history, regular exercise habit, total cholesterol, LDL-cholesterol, eGFR and uric acid), a long duration of chewing betel nut (per 1 year; OR = 1.021; 95% CI = 1.008 to 1.035; *p* = 0.002), old age (*p* < 0.001), alcohol drinking history (*p* = 0.031) and high uric acid (*p* < 0.001) were significantly associated with follow-up MetS in follow-up participants without baseline MetS with a betel nut chewing history. However, cumulative dose (*p* = 0.882) was not significantly associated with follow-up MetS.

Further, regarding MetS components, a long duration of chewing betel nut was significantly associated with follow-up abdominal obesity (per 1 year; OR = 1.012; 95% CI = 1.001 to 1.024; *p* = 0.038) and follow-up hypertriglyceridemia (per 1 year; OR = 1.019; 95% CI = 1.006 to 1.032; *p* = 0.005) in follow-up participants without baseline MetS with a betel nut chewing history, but not with follow-up low HDL-cholesterol (*p* = 0.051), follow-up hyperglycemia (*p* = 0.617) and follow-up high BP (*p* = 0.225).

We further performed comparisons of follow-up MetS, five components of MetS and the numbers of MetS according to quartile of cumulative dose of betel nut chewing in follow-up participants without baseline MetS with a betel nut chewing history (Table S2), and found that there were non-significant trends in the prevalence of follow-up MetS (*p* = 0.446), follow-up abdominal obesity (*p* = 0.719), follow-up hypertriglyceridemia (*p* = 0.796), follow-up low HDL-cholesterol (*p* = 0.354), follow-up hyperglycemia (*p* = 0.884), follow-up high blood pressure (*p* = 0.084) and the numbers of follow-up MetS (*p* = 0.738) corresponding to quartile of cumulative dose of betel nut chewing.

We further evaluated the association between personal habits of alcohol drinking, betel nut chewing and smoking history with baseline MetS, and found there was no significant difference among personal habits with follow-up MetS.

### 3.6. Prevalence of Betel Nut Chewing History and Betel Nut Chewing Years According to the Sum of Components of MetS

Table 6 shows the prevalence of betel nut chewing history and betel nut chewing years according to the sum of components of MetS. There were significant trends for stepwise increases in the prevalence of betel nut chewing history (3.3, 4.8, 6.8, 9.7, 11.5 and 11.4%; *p* < 0.001 for trend) in all participants (*n* = 121,423), duration of betel nut chewing years (12.4, 13.2, 14.4, 15.1, 15.8 and 15.7 years; *p* < 0.001 for trend) in all participants with betel nut chewing history (*n* = 7355), the prevalence of betel nut chewing history (3.8, 5.1, 6.4, 7.6, 8.8 and 7.1 %; *p* < 0.001 for trend) in follow-up participants without baseline MetS (*n* = 21,150), duration of betel nut chewing years (12.7, 14.8, 14.9, 17.5, 17.5 and 15.3 years; *p* = 0.001 for trend) in follow-up participants without baseline MetS with betel nut chewing history (*n* = 1146).

## 4. Discussion

In this study, we examined associations among betel nut chewing with MetS and its components in 121,423 participants at baseline, and in 27,002 after 4 years of follow-up. The results showed that chewing betel nut and a long history of chewing betel nut were associated with MetS and its components at baseline. In addition, chewing betel nut and a long history of chewing betel nut were associated with new-onset MetS and its components at follow-up.

There are several important findings to this study. First, chewing betel nut and a long history of chewing betel nut were associated with MetS at baseline, and new-onset MetS at follow-up. In a cross-sectional study of indigenous Taiwanese conducted in southern Taiwan, chronic betel quid chewing was found to be an independent risk factor for MetS, with adjusted ORs of 1.92 and 1.60 in the male and female betel quid chewers, respectively [35]. In the present study, we also found that betel nut chewers had a higher risk of new-onset MetS. There are several mechanisms for this association. The first mechanism may be due to chronic inflammation [36]. Betel nut chewing has been associated with dental caries and periodontal diseases, both of which lead to chronic inflammation [36]. A study in Pakistan showed that betel nut chewers had significantly higher odds of an elevated C reactive protein (CRP) level as evidence of systemic inflammation (OR 3.23) compared to controls [36]. Another study of patients with oral cancer reported that current and former betel nut users had significantly higher levels of high-sensitivity CRP [36]. Another study showed a higher peripheral leukocyte count in both male and female betel nut chewers, along with a higher plasma TNF-α level in men and higher plasma leptin level in women [35]. The second possible mechanism underlying the relationship between betel nut chewing and MetS may be through increased oxidative stress. Current and former betel nut chewers have been significantly associated with elevated levels of malondialdehyde, a marker of oxidative stress in patients with oral cancer [36]. Therefore, chronic inflammation and oxidative stress may play important roles in the association between betel nut chewing and MetS.

Another important finding of this study is that chewing betel nut and a long history of chewing betel nut were associated with baseline and follow-up abdominal obesity, hypertriglyceridemia and low HDL-cholesterol. Guh et al. reported that the prevalence of MetS in Taiwan from 1993–1996 was around 10%, and that chewing betel nut 10 times/day was associated with MetS, abdominal obesity, hypertriglyceridemia and high BP [26]. In Pakistan, a significant positive association between betel nut chewing and MetS was found in both males (OR = 2.85) and females (OR = 4.22) after adjusting for age and social class. In addition, two of the five components of MetS including low HDL-cholesterol and hypertriglyceridemia were associated with raw betel nut chewing without the use of tobacco [27]. Another population-based study reported an association between betel nut chewing and MetS in men, and significant associations between betel nut chewing and hypertriglyceridemia, abdominal obesity and hyperglycemia [37]. Other epidemiological studies have also shown associations between betel nut chewing and central obesity [26,34] and general obesity [17]. In the present study, we found that abdominal obesity was significantly associated with chewing betel nut and a long history of chewing betel nut. A possible explanation is that areca alkaloids including arecoline, arecaidine, guvacoline and guvacine inhibit the γ-aminobutyric acid (GABA) receptor. The inhibitory effect of betel nut on the GABA receptor may increase appetite resulting in obesity [38,39]. Hypertriglyceridemia was associated with betel nut chewing in our study, which is consistent with previous studies [26,27,37]. Betel nut chewing is often accompanied by other habits such as smoking tobacco and drinking alcohol. Excessive alcohol consumption can increase the levels of both postprandial and fasting serum triglycerides [40]. In an in vitro study, Hsu et al. proposed that betel nut chewing may contribute to an abnormal serum triglyceride level. Arecoline inhibits adipogenic differentiation of preadipocytes and induces adenylyl cyclase-dependent lipolysis. Thus, the authors concluded that arecoline-induced fat cell dysfunction may lead to hyperlipidemia [41]. Further investigations are needed to elucidate the mechanism underlying the association between betel nut chewing and dyslipidemia.

We also found that betel nut chewing was not associated with elevated BP, even after 4 years of follow-up. The influence of betel nut chewing on BP has been inconclusive in previous studies. Yen et al. found that current betel nut chewing was an independent predictive factor for MetS (OR: 1.78) [37]; however, hypertension was not associated with either previous or current betel nut chewing [37]. In addition, Chu reported that new betel nut chewers in Taiwan were associated with an elevated systolic BP, but that this was not seen in habitual chewers [42]. These findings suggest that the effect of elevated BP in betel nut chewers may be transient. Chiou et al. reported that chewing betel nut enhanced sympathetic activity transiently in healthy young adults [43]. In addition, diabetic patients who chew betel nut have been associated with hypertension, especially women [18]. Betel nut chewing appears to activate sympathetic tone, thereby inducing the secretion of catecholamines [38]. Further studies are therefore needed to elucidate the long-term effect of betel nut chewing on BP.

In our follow-up study, a history of chewing betel nut in the participants without MetS at baseline was not associated with hyperglycemia. In other words, even though these participants had chewed betel nut for a long time, they did not have a significantly higher fasting blood sugar level. There are several possible reasons for this finding. First, when preparing betel nut, a betel quid is wrapped in Piper betel leaves, which can induce hypoglycemia [44]. Second, betel nut seed ethanol extract has been shown to have an anti-diabetic effect on male rats [45]. However, betel nut nitrosamines were shown to be diabetogenic and able to induce inheritable glucose intolerance in an animal model [24]. In addition, Hsu et al. conducted an in vitro study and found that arecoline attenuated insulin-induced uptake, suggesting a diabetogenic effect of betel nut [41]. Moreover, a large number of genes relevant to type 2 DM, obesity and MetS were found to have significantly changed in a human monocyte cell line incubated with arecoline and its nitrosated products [46]. Therefore, further investigations are needed to study the influence of betel nut chewing on the serum glucose level.

In our study, a high cumulative dose was associated with baseline MetS. However, the cumulative dose of betel nut chewing was not associated with follow-up MetS, which indicated that betel nut chewing might not contribute to the development of MetS in long-term follow-up. Besides this, after exclusion of participants with smoking and/or alcohol drinking history, betel nut chewing history was not associated with follow-up MetS. Subjects with betel nut chewing, in general, may also be smokers and habitual drinkers. The current investigation also indicated that smoking and alcohol drinking are also independently associated with components of metabolic syndrome, their effects seemed to be more evident than betel nut chewing.

To the best of our knowledge, this is the first longitudinal study to investigate associations between chewing betel nut with MetS and its components. The main strengths of the study are the large-scale investigation and follow-up. However, several limitations to the present study should be noted. First, the TWB does not include data on the use of medications such as anti-diabetic agents, antihypertensive medication and lipid-lowering agents, which may also have affected the development or prevention of MetS, fasting glucose, BP and lipid profile. Therefore, the association between betel nut chewing and MetS might be under-estimated. Second, the participants in this study were of Chinese ethnicity, and thus our findings may not be generalizable to other ethnicities. Finally, according to the statistics from the TWB, about 50% of the participants return for follow-up assessments, which may have resulted in sample bias.

In conclusion, our results showed an association between chewing betel nut and a higher risk of MetS as well as its five components at baseline. In the participants without MetS at baseline, chewing betel nut and a long history of chewing betel nut were associated with the development of MetS after 4 years of follow-up. However, a cumulative dose of betel nut chewing was not associated with follow-up MetS. Betel nut chewing cessation programs are important to reduce the incidence of MetS in Taiwan.

## Figures and Tables

**Table 1:** Comparison of clinical characteristics among participants according to baseline MetS.

Characteristics	MetS (−) (*n* = 94,043)	MetS (+) (*n* = 27,380)	*p*
Age (year)	48.8 ± 10.9	53.6 ± 10.1	<0.001
Male gender (%)	33.7	43.7	<0.001
DM (%)	2.5	14.3	<0.001
Hypertension (%)	6.9	30.6	<0.001
Betel nut chewing history (%)	4.8	10.4	<0.001
Smoking history (%)	25.3	33.9	<0.001
Alcohol drinking history (%)	7.5	12.0	<0.001
Regular exercise habits (%)	40.5	40.8	0.33
BMI (kg/m^2^)	23.3 ± 3.3	27.3 ± 3.8	<0.001
Waist circumference (cm)	80.8 ± 9.1	91.9 ± 9.1	<0.001
Laboratory parameters			<0.001
Fasting glucose (mg/dL)	92.0 ± 13.3	109.2 ± 32.6	<0.001
Triglyceride (mg/dL)	92.6 ± 53.2	194.8 ± 146.3	<0.001
Total cholesterol (mg/dL)	194.5 ±34.8	199.6 ± 38.9	<0.001
HDL-cholesterol (mg/dL)	57.6 ± 12.9	44.2 ± 9.5	<0.001
LDL-cholesterol (mg/dL)	120.0 ± 31.1	124.1 ± 33.8	<0.001
eGFR (mL/min/1.73 m^2^)	104.7 ± 23.5	98.4 ± 24.4	<0.001
Uric acid (mg/dL)	5.2 ± 1.3	6.1 ± 1.5	<0.001

Abbreviations. MetS, metabolic syndrome; DM, diabetes mellitus; BMI, body mass index; HDL, high-density lipoprotein; LDL, low-density lipoprotein; eGFR, estimated glomerular filtration rate.

**Table 2:** Association of betel nut chewing history with baseline MetS and its components in all study participants (*n* = 121,423) using multivariable logistic regression analysis.

Variables	Baseline MetS	Baseline Abdominal Obesity	Baseline Hypertriglyceridemia	Baseline Low HDL-Cholesterol	Baseline Hyperglycemia	Baseline High Blood Pressure
	OR (95% CI)	OR (95% CI)	OR (95% CI)	OR (95% CI)	OR (95% CI)	OR (95% CI)
Betel nut chewing history	1.629 (1.535–1.730) **	1.550 (1.465–1.640) **	1.488 (1.399–1.582) **	1.529 (1.435–1.630) **	1.222 (1.151–1.297) **	1.139 (1.075–1.206) **
Age (per 1 year)	1.051 (1.049–1.053) **	1.029 (1.027–1.030) **	1.015 (1.013–1.016) **	1.019 (1.017–1.020) **	1.072 (1.070–1.074) **	1.076 (1.074–1.077) **
Male (vs. female)	0.653 (0.627–0.680) **	0.258 (0.248–0.267) **	1.463 (1.403–1.526) **	0.257 (0.247–0.268) **	1.430 (1.374–1.489) **	1.720 (1.660–1.783) **
Smoking history	1.175 (1.130–1.222) **	1.132 (1.096–1.171) **	1.215 (1.168–1.265) **	1.209 (1.164–1.256) **	1.086 (1.044–1.130) **	0.925 (0.892–0.959) **
Alcohol drinking history	1.052 (0.998–1.108)	1.121 (1.070–1.175) **	0.918 (0.870–0.969) *	0.754 (0.712–0.799) **	1.258 (1.195–1.324) **	1.299 (1.237–1.364) **
Regular exercise habits	0.710 (0.688–0.732) **	0.737 (0.718–0.757) **	0.684 (0.662–0.707) **	0.761 (0.738–0.784) **	0.869 (0.842–0.896) **	0.943 (0.917–0.970) **
Total cholesterol (per 1 mg/dL)	1.002 (1.001–1.003) **	0.989 (0.988–0.990) **	1.042 (1.041–1.043) **	0.954 (0.953–0.955) **	1.000 (0.999–1.000)	1.000 (0.999–1.001)
LDL-cholesterol (per 1 mg/dL)	0.998 (0.9970.999) **	1.016 (1.015–1.017) **	0.966 (0.965–0.967) **	1.042 (1.041–1.044) **	1.001 (1.000–1.002)	1.002 (1.001–1.002) **
eGFR (per 1 mL/min/1.73 m^2^)	1.005 (1.004–1.006) **	1.006 (1.005–1.006) **	1.005 (1.004–1.005) **	1.004 (1.003–1.004) **	1.007 (1.006–1.007) **	1.000 (0.999–1.001)
Uric acid (per 1 mg/dL)	1.690 (1.669–1.712) **	1.611 (1.592–1.629) **	1.639 (1.617–1.661) **	1.532 (1.513–1.551) **	1.249 (1.234–1.265) **	1.338 (1.323–1.353) **

Values expressed as odds ratio (OR) and 95% confidence interval (CI). Abbreviations are the same as in Table 1. Adjusted for age, gender, smoking history, alcohol history, regular exercise habit, total cholesterol, LDL-cholesterol, eGFR and uric acid. * *p* < 0.05; ** *p* < 0.001.

**Table 3:** Association of betel nut chewing years with baseline MetS and its components in study participants with betel nut chewing history (*n* = 7355) using multivariable logistic regression analysis.

Variables	Baseline MetS	Baseline Abdominal Obesity	Baseline Hypertriglyceridemia	Baseline Low HDL-Cholesterol	Baseline Hyperglycemia	Baseline High Blood Pressure
	OR (95% CI)	OR (95% CI)	OR (95% CI)	OR (95% CI)	OR (95% CI)	OR (95% CI)
Betel nut chewing years (per 1 year)	1.008 (1.004–1.013) **	1.006 (1.001–1.010) *	1.011 (1.006–1.016) **	1.012 (1.007–1.017) **	1.002 (0.998–1.007)	0.999 (0.994–1.003)
Age (per 1 year)	1.028 (1.022–1.033) **	1.011 (1.006–1.016) **	0.988 (0.982–0.993) **	0.995 (0.990–1.001)	1.054 (1.048–1.061) **	1.055 (1.049–1.061) **
Male (vs. female)	0.886 (0.660–1.189)	0.343 (0.257–0.459) **	1.734 (1.247–2.411) *	0.515 (0.386–0.687) **	1.587 (1.147–2.195) *	1.669 (1.238–2.250) *
Smoking history	0.985 (0.800–1.213)	0.837 (0.683–1.025)	1.318 (1.048–1.659) *	1.296 (1.028–1.633) *	0.964 (0.777–1.195)	0.814 (0.660–1.004)
Alcohol drinking history	1.117 (1.010–1.235) *	1.059 (0.962–1.167)	0.970 (0.870–1.083)	0.659 (0.591–0.735) **	1.322 (1.1931–0.465) **	1.501 (1.358–1.659) **
Regular exercise habits	0.798 (0.718–0.888) **	0.824 (0.744–0.912) **	0.679 (0.605–0.763) **	0.692 (0.617–0.777) **	0.914 (0.820–1.018)	1.141 (1.028–1.267) *
Total cholesterol (per 1 mg/dL)	1.012 (1.010–1.015) **	1.000 (0.998–1.002)	1.061 (1.057–1.065) **	0.992 (0.990–0.995) **	1.005 (1.003–1.007) **	1.004 (1.002–1.006) **
LDL-cholesterol (per 1 mg/dL)	0.986 (0.983–0.988) **	1.002 (1.000–1.005)	0.944 (0.940–0.948) **	0.996 (0.993–0.999) *	0.995 (0.992–0.997) **	0.995 (0.993–0.998) **
eGFR (per 1 mL/min/1.73 m^2^)	0.999 (0.996–1.001)	1.000 (0.998–1.002)	0.998 (0.995–1.000)	1.000 (0.997–1.002)	1.005 (1.002–1.007) **	0.998 (0.995–1.000)
Uric acid (per 1 mg/dL)	1.258 (1.213–1.305) **	1.251 (1.207–1.296) **	1.241 (1.192–1.291) **	1.203 (1.158–1.250) **	1.022 (0.985–1.060)	1.183 (1.141–1.227) **

Values expressed as odds ratio (OR) and 95% confidence interval (CI). Abbreviations are the same as in Table 1. Adjusted for age, gender, smoking history, alcohol history, regular exercise habit, total cholesterol, LDL-cholesterol, eGFR and uric acid. * *p* < 0.05; ** *p* <0.001.

**Table 4:** Association of betel nut chewing history with follow-up MetS and its components in follow-up participants without baseline MetS (*n* = 21,150) using multivariable logistic regression analysis.

Variables	Follow-Up MetS	Follow-Up Abdominal Obesity	Follow-Up Hypertriglyceridemia	Follow-Up Low HDL-Cholesterol	Follow-Up Hyperglycemia	Follow-Up High Blood Pressure
	OR (95% CI)	OR (95% CI)	OR (95% CI)	OR (95% CI)	OR (95% CI)	OR (95% CI)
Betel nut chewing history	1.352 (1.134–1.612) *	1.197 (1.035–1.384) *	1.326 (1.130–1.556) *	1.345 (1.113–1.626) *	1.153 (0.982–1.353)	0.998 (0.865–1.152)
Age (per 1 year)	1.033 (1.029–1.038) **	1.018 (1.015–1.021) **	0.996 (0.992–1.000)	1.010 (1.006–1.014) **	1.058 (1.053–1.063) **	1.074 (1.070–1.078) **
Male (vs. female)	0.502 (0.446–0.565) **	0.198 (0.181–0.217) **	1.269 (1.137–1.416) **	0.239 (0.213–0.269) **	1.513 (1.361–1.682) **	1.773 (1.624–1.936) **
Smoking history	1.142 (1.017–1.283) *	1.135 (1.041–1.238) *	1.110 (0.996–1.235)	1.144 (1.023–1.280) *	0.973 (0.875–1.083)	0.930 (0.850–1.018)
Alcohol drinking history	1.085 (0.941–1.252)	1.092 (0.978–1.220)	0.960 (0.841–1.096)	0.786 (0.671–0.920) *	1.255 (1.106–1.423) **	1.305 (1.167–1.458) **
Regular exercise habits	0.790 (0.726–0.861) **	0.754 (0.708–0.802) **	0.753 (0.693–0.819) **	0.836 (0.771–0.907) **	0.956 (0.883–1.035)	0.933 (0.874–0.996) *
Total cholesterol (per 1 mg/dL)	0.980 (0.977–0.983) **	0.985 (0.983–0.987) **	1.013 (1.011–1.016) **	0.925 (0.922–0.928) **	0.995 (0.992–0.997) **	0.998 (0.996–1.000)
LDL-cholesterol (per 1 mg/dL)	1.026 (1.023–1.030) **	1.022 (1.019–1.024) **	0.997 (0.994–0.999) *	1.084 (1.079–1.088) **	1.007 (1.004–1.010) **	1.004 (1.002–1.007) **
eGFR (per 1 mL/min/1.73 m^2^)	1.004 (1.002–1.006) **	1.003 (1.002–1.005) **	1.001 (0.999–1.003)	1.003 (1.001–1.005) **	1.006 (1.004–1.007) **	1.001 (1.000–1.003)
Uric acid (per 1 mg/dL)	1.483 (1.430–1.537) **	1.441 (1.401–1.482) **	1.366 (1.319–1.414) **	1.279 (1.235–1.325) **	1.161 (1.123–1.201) **	1.254 (1.219–1.290) **

Values expressed as odds ratio (OR) and 95% confidence interval (CI). Abbreviations are the same as in Table 1. Adjusted for age, gender, smoking history, alcohol history, regular exercise habit, total cholesterol, LDL-cholesterol, eGFR and uric acid. * *p* < 0.05; ** *p* < 0.001.

**Table 5:** Association of betel nut chewing years with follow-up MetS and its components in follow-up participants without baseline MetS with a betel nut chewing history (*n* = 1146) using multivariable logistic regression analysis.

Variables	Follow-Up MetS	Follow-Up Abdominal obesity	Follow-Up Hypertriglyceridemia	Follow-Up Low HDL-Cholesterol	Follow-Up Hyperglycemia	Follow-Up High Blood Pressure
	OR (95% CI)	OR (95% CI)	OR (95% CI)	OR (95% CI)	OR (95% CI)	OR (95% CI)
Betel nut chewing years (per 1 year)	1.021 (1.008–1.035) *	1.012 (1.001–1.024) *	1.019 (1.006–1.032) *	1.015 (1.000–1.030)	1.003 (0.991–1.015)	1.007 (0.996–1.018)
Age (per 1 year)	1.005 (0.988–1.023)	1.005 (0.991–1.019)	0.961 (0.946–0.976) **	0.985 (0.967–1.003)	1.045 (1.028–1.061) **	1.052 (1.038–1.067) **
Male (vs. female)	0.501 (0.198–1.265)	0.152 (0.065–0.358) **	1.101 (0.448–2.706)	0.707 (0.264–1.894)	1.999 (0.656–6.086)	0.859 (0.378–1.952)
Smoking history	1.296 (0.639–2.628)	1.277 (0.706–2.311)	0.735 (0.402–1.342)	0.782 (0.381–1.601)	1.107 (0.581–2.109)	1.194 (0.680–2.096)
Alcohol drinking history	0.909 (0.666–1.241)	1.000 (0.771–1.298)	0.745 (0.556–0.999) *	0.556 (0.396–0.780) *	1.319 (0.993–1.752)	1.456 (1.130–1.878) *
Regular exercise habits	0.776 (0.560–1.075)	0.881 (0.672–1.156)	0.693 (0.509–0.943) *	0.826 (0.580–1.175)	0.971 (0.725–1.301)	1.053 (0.809–1.369)
Total cholesterol (per 1 mg/dL)	1.008 (1.000–1.017)	0.991 (0.983–0.999) *	1.034 (1.025–1.043) **	0.968 (0.956–0.980) **	1.003 (0.995–1.012)	1.004 (0.997–1.001)
LDL-cholesterol (per 1 mg/dL)	0.992 (0.982–1.001)	1.014 (1.005–1.023) *	0.972 (0.962–0.981) **	1.031 (1.017–1.045) **	1.000 (0.991–1.009)	0.998 (0.990–1.006)
eGFR (per 1 mL/min/1.73 m^2^)	0.999 (0.991–1.007)	1.001 (0.994–1.007)	0.996 (0.988–1.003)	1.003 (0.995–1.012)	1.000 (0.992–1.007)	0.998 (0.992–1.005)
Uric acid (per 1 mg/dL)	1.258 (1.117–1.416) **	1.199 (1.084–1.327) **	1.181 (1.056–1.322) *	1.110 (0.976–1.262)	0.927 (0.831–1.034)	1.147 (1.040–1.266) *

Values expressed as odds ratio (OR) and 95% confidence interval (CI). Abbreviations are the same as in Table 1. Adjusted for age, gender, smoking history, alcohol history, regular exercise habit, total cholesterol, LDL-cholesterol, eGFR and uric acid. * *p* < 0.05; ** *p* < 0.001.

**Table 6:** Prevalence of betel nut chewing history and betel nut chewing years according to the sum of components of MetS.

	0	1	2	3	4	5	*p*
All participants (*n* = 121,423)	*n* = 34,569	*n* = 33,492	*n* = 25,982	*n* = 16,697	*n* = 8166	*n* = 2417	
Betel nut chewing history (%)	3.3	4.8	6.8	9.7	11.5	11.4	<0.001
All participants with betel nut chewing history (*n* = 7355)	*n* = 1144	*n* = 1607	*n* = 1759	*n* = 1621	*n* = 936	*n* = 288	
Betel nut chewing years	12.4 ± 10.7	13.2 ± 10.6	14.4 ± 11.3	15.1 ± 11.4	15.8 ± 11.2	15.7 ± 1.0	<0.001
Follow-up participants without baseline MetS (*n* = 21,150)	*n* = 6039	*n* = 6940	*n* = 5173	*n* = 2235	*n* = 651	*n* = 112	
Betel nut chewing history (%)	3.8	5.1	6.4	7.6	8.8	7.1	<0.001
Follow-up participants without baseline MetS with a betel nut chewing history (*n* = 1146)	*n* = 220	*n* = 343	*n* = 321	*n* = 158	*n* = 76	*n* = 28	
Betel nut chewing years	12.7 ± 10.0	14.8 ± 11.0	14.9 ± 11.9	17.5 ± 12.4	17.5 ± 10.8	15.3 ± 13.1	0.001

Abbreviations. MetS, metabolic syndrome.

## Data Availability

The data underlying this study is from the Taiwan Biobank. Due to restrictions placed on the data by the Personal Information Protection Act of Taiwan, the minimal data set cannot be made publicly available. Data may be available upon request to interested researchers. Please send data requests to: Szu-Chia Chen. Division of Nephrology, Department of Internal Medicine, Kaohsiung Medical University Hospital, Kaohsiung Medical University.

## Supplementary Materials

The following supporting information can be downloaded at: https://www.mdpi.com/article/10.3390/nu14051018/s1, Table S1: Comparison of baseline MetS, five components of MetS and the numbers of MetS according to quartile of cumulative dose of betel nut chewing in study participants with betel nut chewing history. Table S2: Comparison of follow-up MetS, five components of MetS and the numbers of MetS according to quartile of cumulative dose of betel nut chewing in follow-up participants without baseline MetS with a betel nut chewing history.
