# Supplementary material for: Betel Nut Chewing Increases the Risk of Metabolic Syndrome and Its Components in a Large Taiwanese Population Follow-Up Study Category: Original Investigation
Source: Nutrients. 2022 Feb 28;14(5):1018. doi: 10.3390/nu14051018 (PMC8912331; doi:10.3390/nu14051018)
Supplement: Supplementary file 1 [file nutrients-14-01018-s001.zip › nutrients-1578684-supplementary.pdf]

Supplementary Table S1. Comparison of baseline MetS, five components of MetS and the numbers of MetS according to quartile of cumulative dose of betel nut chewing in study participants with betel nut chewing history

Baseline variables	Quartile 1	Quartile 2	Quartile 3	Quartile 4	*p*
MetS (%)	34.0	35.4	40.6	46.9	< 0.001
Abdominal obesity (%)	47.1	51.3	52.5	58.6	< 0.001
Hypertriglyceridemia (%)	36.7	36.8	44.0	44.7	0.001
Low HDL-cholesterol (%)	26.0	27.0	32.5	35.4	< 0.001
Hyperglycemia (%)	30.6	32.4	33.1	40.3	0.001
High blood pressure (%)	49.9	49.9	50.1	57.0	< 0.001
Number of MetS	1.9 ± 1.4	2.0 ± 1.4	2.1 ± 1.4	2.4 ± 1.4	< 0.001

Abbreviations. MetS, metabolic syndrome; HDL, high-density lipoprotein.

Supplementary Table S2. Comparison of follow-up MetS, five components of MetS and the numbers of MetS according to quartile of cumulative dose of betel nut chewing in follow-up participants without baseline MetS with a betel nut chewing history

Follow-up variables	Quartile 1	Quartile 2	Quartile 3	Quartile 4	*p*
MetS (%)	14.8	22.9	20.8	20.5	0.446
Abdominal obesity (%)	38.5	40.0	34.9	33.0	0.719
Hypertriglyceridemia (%)	27.0	26.7	32.1	27.3	0.796
Low HDL-cholesterol (%)	13.9	21.0	19.8	13.6	0.354
Hyperglycemia (%)	26.2	29.5	25.5	25.0	0.884
High blood pressure (%)	41.0	41.9	50.0	56.8	0.084
Number of MetS	1.5 ± 1.1	1.6 ± 1.1	1.6 ± 1.2	1.6 ± 1.1	0.738

Abbreviations. MetS, metabolic syndrome; HDL, high-density lipoprotein.
